# Feasibility investigation of allogeneic endometrial regenerative cells

**DOI:** 10.1186/1479-5876-7-15

**Published:** 2009-02-20

**Authors:** Zhaohui Zhong, Amit N Patel, Thomas E Ichim, Neil H Riordan, Hao Wang, Wei-Ping Min, Erik J Woods, Michael Reid, Eduardo Mansilla, Gustavo H Marin, Hugo Drago, Michael P Murphy, Boris Minev

**Affiliations:** 1 The Second Xiangya Hospital, Central South University, Changsha, PR China; 2 Department of Cardiothoracic Surgery, University of Utah, Salt Lake City, USA; 3 Medistem Inc, San Diego, USA; 4 Department of Surgery, University of Western Ontario, Canada; 5 General Biotechnology LLC, Indiana, USA; 6 Body in Motion Consulting, Kitchener, Canada; 7 Burns Hospital, Buenos Aires City, Argentina; 8 Division of Vascular Surgery, Indiana University School of Medicine, Indiana, USA; 9 Moores Cancer Center, University of California, San Diego; 10 Division of Neurosurgery, University of California San Diego, San Diego, USA

## Abstract

Endometrial Regenerative Cells (ERC) are a population of mesenchymal-like stem cells having pluripotent differentiation activity and ability to induce neoangiogenesis. In vitro and animal studies suggest ERC are immune privileged and in certain situations actively suppress ongoing immune responses. In this paper we describe the production of clinical grade ERC and initial safety experiences in 4 patients with multiple sclerosis treated intravenously and intrathecally. The case with the longest follow up, of more than one year, revealed no immunological reactions or treatment associated adverse effects. These preliminary data suggest feasibility of clinical ERC administration and support further studies with this novel stem cell type.

## Introduction

Endometrial Regenerative Cells (ERC) are a population of plastic adherent, mesenchymal-like stem cells that are possess in vitro pluripotency, and in vivo therapeutic activity in models of limb ischemia and infarcts [1-4]. Phenotypically ERC appear to share some markers with mesenchymal stem cells such as CD90 and CD105 but are unique in that they express hTERT and OCT-4 [1,2]. Immunological characterization of ERC revealed hypoimmunogenicity when used as stimulators in mixed lymphocyte reaction, as well as active suppression of proliferating T cells in vitro. In vivo ERC appear to induce therapeutic effects in immune competent xenogeneic recipients [4]. Thus theoretically ERC may be useful as an allogeneic "off-the-shelf" therapy.

The use of allogeneic cells as a therapeutic approach in immune competent recipients has previously been performed with bone marrow derived mesenchymal stem cells (MSC) which are known to inhibit ongoing mixed lymphocyte reaction (MLR) [5], induce generation of T regulatory cells [6], and suppress autoimmunity *in vivo *in conditions such as collagen induced arthritis [7] and experimental allergic encephalomyelitis [8]. In animal models, acceleration of wound healing [9], or post-infarct recovery [10], has been accomplished by administration of allogeneic mesenchymal cells.

Allogeneic MSC therapy is a clinical reality. For example, cord blood derived MSC have also demonstrated benefit in a patients with critical limb ischemia caused by Buerger's Disease [11]. Allogeneic bone marrow derived MSC have been used by academic investigators for treatment of diseases such as graft versus host (GVHD) [12-17], osteogenesis imperfecta [18], Hurler syndrome, metachromatic leukodystrophy [19], and acceleration of hematopoietic stem cell engraftment [20-22] with clinical benefit. The company Osiris Therapeutics has successfully completed Phase I safety studies using allogeneic MSCs and has currently ongoing Phase II and Phase III trials for Type I Diabetes, Crohn's Disease, and Graft Versus Host Disease using allogeneic bone marrow derived MSC [23]. Intravenous administration of allogeneic MSCs by Osiris was also reported to induce a statistically significant improvement of cardiac function of MI patients in a double-blind study [24]. Other companies have entered clinical trials using allogeneic MSC-based products. Athersys is currently in Phase I trials using its MultiStem™ technology, which involves *ex vivo *expanded multipotent adult progenitor cells (MAPC) for post-infarct heart repair [25]. Angioblast Systems has recently announced initiation of Phase II trials using Mesenchymal Precursor Cells™ for stimulation of cardiac angiogenesis [26]. Neuronyx is currently performing Phase I clinical trials using allogeneic human adult bone marrow-derived somatic cells (*hABM-SC*) for post infarct healing [27].

Given the general clinical safety profile of MSC from other sources, we conducted initial studies to determine the safety profile of ERC. We have previously demonstrated karyotypical stability up to 68 doublings [1], as well as lack of tumor formation ability or tumor acceleration in animal models [4]. In this short report we detail expansion, quality control, and initial safety data from patients treated under compassionate use in a physician-initiated setting. A detailed description of the therapeutic effects and rationale for use in the indications described will be provided in subsequent publications.

## Methods

### Patients

Four patients were treated as part of a compassionate use, physician initiated program. All patients have been accepted by an independent medical review board deeming that the patients have failed all standard treatment options. Additionally, local IRB approval for the general protocols and procedures is in place. All patients were not allergic to penicillin or ciprofloxacin.

### Donors

Donors were selected after rigorous testing according to federal regulation 21 CFR1271 regarding allogeneic cell products. Specifically, healthy, non-smoking, female volunteers between 18–30 years of age signed informed consent form for providing menstrual blood sample. The volunteers underwent a standard medical history and physical examination, as well as evaluation for malignancy, diabetes, heart disease, in addition to CBC and metabolic panel. Only donors negative for HIV-1, HIV-2, HTLV-II, hepatitis B surface antigen, hepatitis B core antigen, hepatitis C, VDRL, papilloma virus and trypanosome cruzi were allowed to participate in this study.

### Collection

Before the collection procedure a "collection tube" was prepared in a class 100 Biological Safety Cabinet located in a Class 10,000 Clean Room. To prepare the collection tube, 0.2 ml amphotericin B (Sigma-Aldrich, St Louis, MO), 0.2 ml penicillin/streptomycin (Sigma) and 0.1 ml EDTA-Na2 (Sigma) were added to a 50 ml conical tube containing 30 ml of GMP-grade phosphate buffered saline (PBS). Collection of 5 ml of menstrual blood was performed according to a modification of our published procedure [1]. Collection was performed by the donor. A sterile Diva cup was inserted into the vagina and left in place for 30–60 minutes. After removal, the contents of the Diva cup were decanted into the collection tube. The collection tube was then taken to the clean room where it was centrifuged at 600 g for 10 minutes. The collection tube was then transported to the Biological Safety Cabinet where the supernatant was removed, and the tube was topped up to 50 ml with PBS in the Biological Safety Cabinet and cells were washed by centrifugation at 600 g for 10 minutes at room temperature. The cell pellet was washed 3 times with 50 ml of PBS, and mononuclear cells were collected by Ficoll-Paque (Fisher Scientific, Portsmouth NH) density gradient. Mononuclear cells were washed 3 times in PBS and resuspended in 5 ml complete DMEM-low glucose medium (GibcoBRL, Grand Island, NY) supplemented with 10% Fetal Bovine Serum selected lots having endotoxin level < = 10 EU/ml, and hemoglobin level < = 25 mg/dl clinical grade ciprofloxacin (5 mg/mL, Bayer A.G., Germany) and 4 mM L-glutamine (cDMEM). The serum lot used was sequestered and one lot was used for all experiments. The resulting cells were mononuclear cells substantially free of erythrocytes and polymorphonuclear leukocytes as assessed by visual morphology microscopically. Viability of the cells was assessed using a Guava EasyCyte Mini flow cytometer, Viacount reagents, Cytosoft Software version 4.2.1, Guava Technologies, inc. Hayward, CA (Guava flow cytometer). Only samples with > 90% viability were selected for culture.

### Expansion

Cells were plated in a T-75 flask containing 15 ml of cDMEM and cultured for 24 hours at 37°C at 5% CO2 in a fully humidified atmosphere. This allows the ERC precursors to adhere. Non-adherent cells were washed off using cDMEM by gentle rinsing of the flask. Adherent cells were subsequently detached by washing the cells with PBS and addition of 0.05% trypsin containing EDTA (Gibco, Grand Island, NY, USA) for 2 minutes at 37°C at 5% CO2 in a fully humidified atmosphere. Cells were centrifuged, washed and plated in T-175 flask in 30 ml of cDMEM. This results in approximately 10,000 ERC per initiating T-175 flask. The flask was then cultured for 5 days which yields approximately 1 million cells in the T-175 flask (*passage 1*). Subsequently cells were passaged at approximately 200,000 cells in a T-175 flask. At passage 3–4, approximately 100–200 million cells were harvested.

### Characterization and release criteria

Cells aliquots from each donor batch have met the following release criteria: (i) negative for bacterial and mycoplasma contamination; (ii) endotoxin levels < 1.65 EU/ml; (iii) morphology consistent with adherent, fibroblastic-like shape; (iv) CD90 and CD105 positive (> 90%) and CD45 and CD34 negative (< 5%) by flow cytometry; (v) Cell viability > 95% by trypan blue staining and Guava flow cytometer. In addition, karyotypic normality of the cells was also assessed by an independent laboratory for each batch.

### Administration

Intravenous administration was performed by intravenous injection using USP-grade saline and autologous heat inactivated serum (50%). Administration time was 10 minutes approximately 1 million cells/ml were injected. For intrathecal injection, 6 million ERC's cells in USP-grade clinical normal saline (Baxter) and autologous heat inactivated serum (50%) were drawn in a 10 ml syringe. The syringe was attached to the lumbar puncture needle. In order to ensure the needle was still in the CSF, the plunger was drawn back to aspirate a small volume of CSF. The volume of 6 ml was injected slowly with the patient repeatedly asked if there was pain during the injection process. At no time was there resistance in the procedure. Once the cell solution has been injected into the CSF the plunger of the syringe was kept fully depressed and the syringe and lumbar puncture needle removed together. The injection site and general condition of the patient was monitored for 30 minutes after the first and each subsequent administration at the hospital to look for a possible allergic reaction.

### Case reports

The patients were treated as part of a compassionate use, physician initiated program. Since patients received other medical interventions and therapies in addition to ERC, only safety parameters will be discussed in this report. Summarized details of the patients and treatments are provided in Table 1. Overall safety evaluations performed are depicted in Table 2.

**Table 1:** Summary of Patients Treated

**Patient**	**Condition**	**Route**	**Total Injected**	**Follow Up**	**Notable Events**
AA	MS	IV & IT	16 million	12 months	None

PW	MS	IT	30 million	2 months	None

RH	MS	IT	30 million	2 months	None

JU	MS	IT	30 million	5 months	None

**Table 2:** Safety Parameters

**Patient**	**Physical Exam**	**CBC/Biochem Panel**	**Fecal Occult Blood**	**Chest X-Ray**	**PSA, CEA, alpha fetoprotein**
AA	X	X	X	X	X

PW	X	X	X	X	X

RH	X	X	ND	X	X

JU	X	X	ND	ND	X

### Patient 1: Multiple Sclerosis (AA) Intravenous and Intrathecal

This 47-year-old patient was diagnosed with multiple sclerosis in November 2000. Due to severe pain in the left arm and right leg refractory to medication, as well as fatigue and impaired mobility, the patient sought non-conventional treatment options in December 2006. The patient presented in July of 2007. After being explained the experimental nature of the proposed procedure, informed consent was obtained. Administration of 3 million ERC was performed intravenously on days 1, 3, and 4; on day 2 she received an intrathecal injection with ERC's. No adverse reactions were noted at the time of administration. On July 24, 2008, the patient returned for a follow-up examination and requested additional ERC treatment. This opportunity was used to perform a physical exam, chest x-ray, complete blood count, serum biochemistry, CEA, alpha-fetoprotein, fecal occult blood. All of these tests generated no evidence of abnormality. The physical exam emphasized the injection site, which revealed no inflammation, masses or abnormalities. Telephone interview with the patient on December 2008 revealed no notable events or abnormities.

### Patient 2: Multiple Sclerosis: (PW) Intrathecal

According to his neurologist, this 39-year-old patient first started noting signs of fatigue in 1995, with staggering gait and cognitive decline. The patient had never experienced any relapse remitting type of presentation. The patient presented in May 2008, requesting experimental stem cell therapy. After being explained the nature of the procedure and possible adverse effects, the patient signed an informed consent form. Administration of 5 intrathecal injections of 6 million ERC was performed on days 1, 3, 6, 8, and 10. Examination of the injection area was made prior to subsequent injections and release of the patient. No inflammatory lesions or abnormalities were observed. Importantly, physical and neurological examination did not reveal abnormalities, or inflammatory lesions at injection site. Complete blood counts and serum biochemistry was unremarkable as of July 22, 2008. Telephone interview with the patient on December 2008 revealed no notable events or abnormities.

### Patient 3: Multiple Sclerosis: (RH) Intrathecal

This 53-year-old male patient was diagnosed with Relapsing-remitting MS in 2005. In May 2008, the patient was treated with five intrathecal infusions of 6 million ERC. All infusions were performed within a 9-day period and were very well tolerated without any significant side effects. During the infusions we observed no adverse or side effects. No local or systemic effects were noted. After each infusion the patient was observed for 15 to 20 minutes to look for a possible allergic reaction, but no such reaction was noted. In September 2008, the patient underwent a panel of post-treatment medical evaluation tests, including CBC, stool culture, basic metabolic panel, liver function panel, CEA and PSA. All tests revealed no abnormalities. Telephone interview with the patient on December 2008 revealed no notable events or abnormities.

### Patient 4 Multiple Sclerosis (JU) Intrathecal

This 36-year-old male patient was diagnosed with Relapsing-remitting MS in September of 1993. His presenting symptoms in January of 1993 were noticeable tingling and burning sensation in the right leg, followed by lower body paralysis lasting almost three weeks. In 2007 he was treated with Tysabri (Natalizumab, Biogen Idec) for 6 months without success. The patient's condition deteriorated significantly and he was immobilized most of the time with severe pain in the coccygeal area, significantly impacted balance and coordination, very low energy level, heat sensitivity, bowel and bladder function difficulties, and substantial fatigue and depression. Although the pain was treated well with Carbamazepine, the patient was on full disability in 2007 and the first half of 2008. In June of 2008, the patient was treated with five intrathecal infusions of 6 million ERC. All infusions were performed within a 9-day period and were very well tolerated without any significant side effects. The only noted side effect was mild self-limiting headache, a common side effect of lumbar puncture (Reference Feron). After each infusion the patient was observed for 15 to 20 minutes to look for a possible allergic reaction, but no such reaction was noted. In August 2008, the patient underwent a physical examination and several post-treatment evaluation tests, including CBC, basic metabolic panel, liver function panel, CEA and PSA. All tests revealed no abnormalities. PA and lateral chest X-ray views revealed normal findings with a minimal patchy lingular atelectasis. Telephone interview with the patient on December 2008 revealed no notable events or abnormities.

## Discussion

In this study we demonstrated for the first time feasibility of administration of ERC-based cell therapy in four patients with MS. This "off-the-shelf" allogeneic ERC therapy could conceptually have several positive aspects such as: a) ease of administration; b) ability to use optimized cells; and c) administration of multiple doses. The most clinically advanced form of stem cell therapy, hematopoietic stem cells, either extracted from bone marrow by iliac crest puncture, or by G-CSF mobilization, has demonstrated varying degrees of efficacy in conditions such as heart failure [28,29], liver failure [30,31], peripheral artery disease [32-35], and spinal cord injury [36-38]. The effects of bone marrow stem cell-based treatments appear to be primarily due to trophic support through secretion of various growth factors [39,40], stimulation of angiogenesis [41], and possibly fusion/transdifferentiation [42-44], although this latter possibility is quite controversial [45]. Unfortunately, autologous approaches are limited by the considerable inter-individual heterogeneity in the stem cell activity. For example, older patients are known to have reduced bone marrow stem cell regenerative activity, as well as lower angiogenic potential, as compared to younger people [46]. Additionally, patients with cardiovascular risk factors have severely compromised regenerative potential compared to age-matched controls [47]. Accordingly, an "off-the-shelf", standardized stem cell population would be a much more attractive treatment alternative for a variety of immunomodulatory and regenerative indications.

The concept of "off-the-shelf" stem cell therapies has been clinically performed in trials using bone marrow derived MSC. The extensive clinical experience with these cells demonstrates no evidence of adverse effects in over 10 clinical trials to date [12-22]. Given that ERC are derived from the endometrium, we suggested that these cells might be able to support the angiogenesis in the model of critical limb ischemia [4]. Our preliminary results demonstrate superior angiogenic potential of ERC compared to bone marrow derived MSC, and our published data showed superior growth factor production as compared to placental MSC [1]. Additionally, it has been reported that ERC and ERC-like cells are capable of differentiating into 9 different tissues including cardiac, hepatic, pancreatic, bone, adipose, cartilage, endothelial, neural, and pulmonary tissues [1,2]. In contrast, freshly isolated bone marrow derived MSC do not appear to possess such pluripotency unless extensively manipulated ex vivo. Therefore, there is a possibility that ERC may be useful for numerous clinical indications. Hida et al demonstrated in vivo cardiac repair using a menstrual blood derived cell type possessing some similarity to the ERC [3].

ERC possess various characteristics similar to MSC including ability to immune modulate [4] and induce Treg production [48]. MSC have been previously demonstrated to inhibit induction and progression of experimental allergic encephalomyelitis (EAE), a rodent model of multiple sclerosis [8]. Furthermore, introduction of MSC intrathecally has been reported by Slavin's group to mediate beneficial effects in pilot trials in patients with neurodegenerative diseases including MS [49]. One of the goals of this report is to propose the possibility of using ERC as a substitute for bone marrow MSC given that ERC appear to be more practical in terms of expansion and maintenance of karyotypic stability.

## Conclusion

We describe the first clinical use of allogeneic ERC. With the caveat of a small sample size and limited number of injections, it appears that ERC may be administered intravenously and intrathecally without immediate immunological reactions or ectopic tissue formation. Although in this study we report on safety of the cells, we should note that disease progression did not occur in the patients treated as reported by their neurologists, based on radiological and functional assessment. We suggest further clinical investigation of ERC is warranted.

## Consent

Written informed consent was obtained from the patients for publication of these case reports.

## Completing interests

Thomas Ichim and Neil H Riordan are management and shareholders of Medistem Inc, a company that has filed an IND and owns intellectual property related to ERC.

## Authors' contributions

All authors read and approved the final manuscript. ZZ, ANP, TEI, NHR, HW, WM, EJM, MR, EM, GHM, HD, MPM, and BM conceived experiments, interpreted data, and wrote the manuscript.

## References

[B1] Meng X, Ichim TE, Zhong J, Rogers A, Yin Z, Jackson J, Wang H, Ge W, Bogin V, Chan KW (2007). Endometrial regenerative cells: a novel stem cell population. J Transl Med.

[B2] Patel AN, Silva F (2008). Menstrual blood stromal cells: the potential for regenerative medicine. Regen Med.

[B3] Hida N, Nishiyama N, Miyoshi S, Kira S, Segawa K, Uyama T, Mori T, Miyado K, Ikegami Y, Cui C (2008). Novel cardiac precursor-like cells from human menstrual blood-derived mesenchymal cells. Stem Cells.

[B4] Murphy MP, Wang H, Patel AN, Kambhampati S, Angle N, Chan K, Marleau AM, Pyszniak A, Carrier E, Ichim TE, Riordan NH (2008). Allogeneic endometrial regenerative cells: an "Off the shelf solution" for critical limb ischemia?. J Transl Med.

[B5] Deng W, Han Q, Liao L, Li C, Ge W, Zhao Z, You S, Deng H, Zhao RC (2004). Allogeneic bone marrow-derived flk-1+Sca-1-mesenchymal stem cells leads to stable mixed chimerism and donor-specific tolerance. Exp Hematol.

[B6] Prevosto C, Zancolli M, Canevali P, Zocchi MR, Poggi A (2007). Generation of CD4+ or CD8+ regulatory T cells upon mesenchymal stem cell-lymphocyte interaction. Haematologica.

[B7] Augello A, Tasso R, Negrini SM, Cancedda R, Pennesi G (2007). Cell therapy using allogeneic bone marrow mesenchymal stem cells prevents tissue damage in collagen-induced arthritis. Arthritis Rheum.

[B8] Zappia E, Casazza S, Pedemonte E, Benvenuto F, Bonanni I, Gerdoni E, Giunti D, Ceravolo A, Cazzanti F, Frassoni F (2005). Mesenchymal stem cells ameliorate experimental autoimmune encephalomyelitis inducing T-cell anergy. Blood.

[B9] Wu Y, Chen L, Scott PG, Tredget EE (2007). Mesenchymal stem cells enhance wound healing through differentiation and angiogenesis. Stem Cells.

[B10] Imanishi Y, Saito A, Komoda H, Kitagawa-Sakakida S, Miyagawa S, Kondoh H, Ichikawa H, Sawa Y (2008). Allogenic mesenchymal stem cell transplantation has a therapeutic effect in acute myocardial infarction in rats. J Mol Cell Cardiol.

[B11] Kim SW, Han H, Chae GT, Lee SH, Bo S, Yoon JH, Lee YS, Lee KS, Park HK, Kang KS (2006). Successful stem cell therapy using umbilical cord blood-derived multipotent stem cells for Buerger's disease and ischemic limb disease animal model. Stem Cells.

[B12] Le Blanc K, Frassoni F, Ball L, Locatelli F, Roelofs H, Lewis I, Lanino E, Sundberg B, Bernardo ME, Remberger M (2008). Mesenchymal stem cells for treatment of steroid-resistant, severe, acute graft-versus-host disease: a phase II study. Lancet.

[B13] Ning H, Yang F, Jiang M, Hu L, Feng K, Zhang J, Yu Z, Li B, Xu C, Li Y (2008). The correlation between cotransplantation of mesenchymal stem cells and higher recurrence rate in hematologic malignancy patients: outcome of a pilot clinical study. Leukemia.

[B14] Ball L, Bredius R, Lankester A, Schweizer J, Heuvel-Eibrink M van den, Escher H, Fibbe W, Egeler M (2008). Third party mesenchymal stromal cell infusions fail to induce tissue repair despite successful control of severe grade IV acute graft-versus-host disease in a child with juvenile myelo-monocytic leukemia. Leukemia.

[B15] Ringden O, Uzunel M, Rasmusson I, Remberger M, Sundberg B, Lonnies H, Marschall HU, Dlugosz A, Szakos A, Hassan Z (2006). Mesenchymal stem cells for treatment of therapy-resistant graft-versus-host disease. Transplantation.

[B16] Le Blanc K, Rasmusson I, Sundberg B, Gotherstrom C, Hassan M, Uzunel M, Ringden O (2004). Treatment of severe acute graft-versus-host disease with third party haploidentical mesenchymal stem cells. Lancet.

[B17] Muller I, Kordowich S, Holzwarth C, Isensee G, Lang P, Neunhoeffer F, Dominici M, Greil J, Handgretinger R (2008). Application of multipotent mesenchymal stromal cells in pediatric patients following allogeneic stem cell transplantation. Blood Cells Mol Dis.

[B18] Horwitz EM, Gordon PL, Koo WK, Marx JC, Neel MD, McNall RY, Muul L, Hofmann T (2002). Isolated allogeneic bone marrow-derived mesenchymal cells engraft and stimulate growth in children with osteogenesis imperfecta: Implications for cell therapy of bone. Proc Natl Acad Sci USA.

[B19] Koc ON, Day J, Nieder M, Gerson SL, Lazarus HM, Krivit W (2002). Allogeneic mesenchymal stem cell infusion for treatment of metachromatic leukodystrophy (MLD) and Hurler syndrome (MPS-IH). Bone Marrow Transplant.

[B20] Le Blanc K, Samuelsson H, Gustafsson B, Remberger M, Sundberg B, Arvidson J, Ljungman P, Lonnies H, Nava S, Ringden O (2007). Transplantation of mesenchymal stem cells to enhance engraftment of hematopoietic stem cells. Leukemia.

[B21] Lazarus HM, Koc ON, Devine SM, Curtin P, Maziarz RT, Holland HK, Shpall EJ, McCarthy P, Atkinson K, Cooper BW (2005). Cotransplantation of HLA-identical sibling culture-expanded mesenchymal stem cells and hematopoietic stem cells in hematologic malignancy patients. Biol Blood Marrow Transplant.

[B22] Ball LM, Bernardo ME, Roelofs H, Lankester A, Cometa A, Egeler RM, Locatelli F, Fibbe WE (2007). Cotransplantation of ex vivo expanded mesenchymal stem cells accelerates lymphocyte recovery and may reduce the risk of graft failure in haploidentical hematopoietic stem-cell transplantation. Blood.

[B23] Osiris Theraputics ltd. http://www.osiris.com.

[B24] Osiris Therapeutics Osiris Therapeutics Announces Positive Results in Groundbreaking Stem Cell Trial to Treat Heart. http://www.osiris.com/products_provacel.php.

[B25] Athersys Inc. Athersys IND for MultiStem Authorized for Phase I Clinical Trial in Bone Marrow Transplant Support. http://ir.athersys.com/releasedetail.cfm?ReleaseID=276046.

[B26] Angioblast systems United States FDA clears phase 2 trial for congestive heart failure. http://www.angioblast.com/news/pressrelease10.pdf.

[B27] Garnet Biotheraputics. http://www.neuronyx.com/therapeutic_targets.php.

[B28] Abdel-Latif A, Bolli R, Tleyjeh IM, Montori VM, Perin EC, Hornung CA, Zuba-Surma EK, Al-Mallah M, Dawn B (2007). Adult bone marrow-derived cells for cardiac repair: a systematic review and meta-analysis. Arch Intern Med.

[B29] Schachinger V, Erbs S, Elsasser A, Haberbosch W, Hambrecht R, Holschermann H, Yu J, Corti R, Mathey DG, Hamm CW (2006). Improved clinical outcome after intracoronary administration of bone-marrow-derived progenitor cells in acute myocardial infarction: final 1-year results of the REPAIR-AMI trial. Eur Heart J.

[B30] Levicar N, Pai M, Habib NA, Tait P, Jiao LR, Marley SB, Davis J, Dazzi F, Smadja C, Jensen SL (2008). Long-term clinical results of autologous infusion of mobilized adult bone marrow derived CD34+ cells in patients with chronic liver disease. Cell Prolif.

[B31] Khan AA, Parveen N, Mahaboob VS, Rajendraprasad A, Ravindraprakash HR, Venkateswarlu J, Rao SG, Narusu ML, Khaja MN, Pramila R (2008). Safety and efficacy of autologous bone marrow stem cell transplantation through hepatic artery for the treatment of chronic liver failure: a preliminary study. Transplant Proc.

[B32] Van Huyen JP, Smadja DM, Bruneval P, Gaussem P, Dal-Cortivo L, Julia P, Fiessinger JN, Cavazzana-Calvo M, Aiach M, Emmerich J (2008). Bone marrow-derived mononuclear cell therapy induces distal angiogenesis after local injection in critical leg ischemia. Mod Pathol.

[B33] Tateishi-Yuyama E, Matsubara H, Murohara T, Ikeda U, Shintani S, Masaki H, Amano K, Kishimoto Y, Yoshimoto K, Akashi H (2002). Therapeutic angiogenesis for patients with limb ischaemia by autologous transplantation of bone-marrow cells: a pilot study and a randomised controlled trial. Lancet.

[B34] Gu YQ, Zhang J, Guo LR, Qi LX, Zhang SW, Xu J, Li JX, Luo T, Ji BX, Li XF (2008). Transplantation of autologous bone marrow mononuclear cells for patients with lower limb ischemia. Chin Med J (Engl).

[B35] Van Tongeren RB, Hamming JF, Fibbe WE, Van Weel V, Frerichs SJ, Stiggelbout AM, Van Bockel JH, Lindeman JH (2008). Intramuscular or combined intramuscular/intra-arterial administration of bone marrow mononuclear cells: a clinical trial in patients with advanced limb ischemia. J Cardiovasc Surg (Torino).

[B36] Chernykh ER, Stupak VV, Muradov GM, Sizikov MY, Shevela EY, Leplina OY, Tikhonova MA, Kulagin AD, Lisukov IA, Ostanin AA, Kozlov VA (2007). Application of autologous bone marrow stem cells in the therapy of spinal cord injury patients. Bull Exp Biol Med.

[B37] Sykova E, Homola A, Mazanec R, Lachmann H, Konradova SL, Kobylka P, Padr R, Neuwirth J, Komrska V, Vavra V (2006). Autologous bone marrow transplantation in patients with subacute and chronic spinal cord injury. Cell Transplant.

[B38] Yoon SH, Shim YS, Park YH, Chung JK, Nam JH, Kim MO, Park HC, Park SR, Min BH, Kim EY (2007). Complete spinal cord injury treatment using autologous bone marrow cell transplantation and bone marrow stimulation with granulocyte macrophage-colony stimulating factor: Phase I/II clinical trial. Stem Cells.

[B39] Pallante BA, Edelberg JM (2006). Cell sources for cardiac regeneration – which cells and why. Am Heart Hosp J.

[B40] Imberti B, Morigi M, Tomasoni S, Rota C, Corna D, Longaretti L, Rottoli D, Valsecchi F, Benigni A, Wang J (2007). Insulin-like growth factor-1 sustains stem cell mediated renal repair. J Am Soc Nephrol.

[B41] Tse HF, Lau CP (2007). Therapeutic angiogenesis with bone marrow – derived stem cells. J Cardiovasc Pharmacol Ther.

[B42] Khurana S, Mukhopadhyay A (2008). In vitro transdifferentiation of adult hematopoietic stem cells: An alternative source of engraftable hepatocytes. J Hepatol.

[B43] Dooner MS, Aliotta JM, Pimentel J, Dooner GJ, Abedi M, Colvin G, Liu Q, Weier HU, Johnson KW, Quesenberry PJ (2008). Conversion potential of marrow cells into lung cells fluctuates with cytokine-induced cell cycle. Stem Cells Dev.

[B44] Yoon J, Choi SC, Park CY, Choi JH, Kim YI, Shim WJ, Lim DS (2008). Bone marrow-derived side population cells are capable of functional cardiomyogenic differentiation. Mol Cells.

[B45] Wagers AJ, Sherwood RI, Christensen JL, Weissman IL (2002). Little evidence for developmental plasticity of adult hematopoietic stem cells. Science.

[B46] Dimmeler S, Leri A (2008). Aging and disease as modifiers of efficacy of cell therapy. Circ Res.

[B47] Schmidt-Lucke C, Rossig L, Fichtlscherer S, Vasa M, Britten M, Kamper U, Dimmeler S, Zeiher AM (2005). Reduced number of circulating endothelial progenitor cells predicts future cardiovascular events: proof of concept for the clinical importance of endogenous vascular repair. Circulation.

[B48] Meng I, Riordan Pct Patent Application (WO/2008/148105) Endometrial Stem Cells And Methods of Making and Using Same. http://www.wipo.int/pctdb/en/wo.jsp?WO=2008148105.

[B49] Karussis D, Kassis I, Kurkalli BG, Slavin S (2008). Immunomodulation and neuroprotection with mesenchymal bone marrow stem cells (MSCs): a proposed treatment for multiple sclerosis and other neuroimmunological/neurodegenerative diseases. J Neurol Sci.

